# Impact of Education and Network for Avian Influenza H5N1 in Human: Knowledge, Clinical Practice, and Motivation on Medical Providers in Vietnam

**DOI:** 10.1371/journal.pone.0030384

**Published:** 2012-01-23

**Authors:** Toshie Manabe, Pham Thi Phuong Thuy, Koichiro Kudo, Vu Thi Tuong Van, Jin Takasaki, Nguyen Dang Tuan, Dao Xuan Co, Dang Hung Minh, Shinyu Izumi, Nguyen Gia Binh, Ngo Quy Chau, Tran Thuy Hanh

**Affiliations:** 1 National Center for Global Health and Medicine, Tokyo, Japan; 2 NCGM-BMH Medical Collaboration Center, Hanoi, Vietnam; 3 Bach Mai Hospital, Hanoi, Vietnam; The University of Hong Kong, China

## Abstract

**Background:**

Knowledge, clinical practice, and professional motivation of medical providers relating to H5N1 infection have an important influence on care for H5N1 patients who require early diagnosis and early medical intervention.

**Methods/Principal Findings:**

Novel educational programs including training and workshops for medical providers relating to H5N1 infection in Vietnam were originally created and implemented in 18 provincial hospitals in northern Vietnam between 2008 and 2010. A self-administered, structured questionnaire survey was conducted in 8 provincial hospitals where both educational training and workshops were previously provided. A total of 326 medical providers, including physicians, nurses, and laboratory technicians who attended or did not attend original programs were enrolled in the survey. Knowledge, clinical attitudes and practice (KAP), including motivation surrounding caring for H5N1 patients, were evaluated. The study indicated a high level of knowledge and motivation in all professional groups, with especially high levels in laboratory technicians. Conferences and educational programs were evaluated to be the main scientific information resources for physicians, along with information from colleagues. The chest radiographs and the initiation of antiviral treatment in the absence of RT-PCR result were identified as gaps in education. Factors possibly influencing professional motivation for caring for H5N1 patients included healthcare profession, the hospital where the respondents worked, age group, attendance at original educational programs and at educational programs which were conducted by international health-related organizations.

**Conclusions:**

Educational programs provide high knowledge and motivation for medical providers in Vietnam caring for H5N1 patients. Additional educational programs related to chest radiographs and an initiation of treatment in the absence of RT-PCR are needed. Networking is also necessary for sharing updated scientific information and practical experiences. These enhanced KAPs by educational programs and integrated systems among hospitals should result in appropriate care for H5N1 patients and may reduce morbidity and mortality.

## Introduction

Avian influenza (H5N1) infection in human is associated with a high mortality rate [1]. One of the main causes may be delayed diagnosis and delayed initiation of antiviral administration [2], [3]. Previously, a Knowledge, Attitude, and Practice (KAP) survey was conducted in people living in H5N1 high-risk communities in Vietnam; awareness and behaviours relating to H5N1 infection and relating to the importance of receiving early medical assistance was evaluated [4]. However, even if patients with H5N1 infection visit medical facilities at the early stage of their illness, a delay in diagnosis or initiation of antiviral drug administration can occur due to a lack of medical resources or insufficient knowledge or experience of medical providers who treat H5N1 patients. Although Vietnam has experienced a relatively high prevalence of H5N1 infection compared to other countries, only 26 has been reported since 2007. This describes that only small number of medical providers have experiences for treating H5N1 patients in Vietnam. Professional motivation on the part of medical providers is also required for treating patients with this fatal illness, for which human-to-human transmission has not yet been evidenced [5]. KAP and motivation relating to H5N1 in hospital-based medical providers in high-risk areas have a strong impact on patients' survival; however, KAP and motivation have not yet been studied in Vietnam. The aim of this study was to assess KAP and motivation relating to H5N1 infection in medical providers in Vietnam in order to enhance their roles in clinical practice and to improve the survival rate for H5N1 infections.

## Materials and Methods

### Study sites and subjects

**Figure 1:**
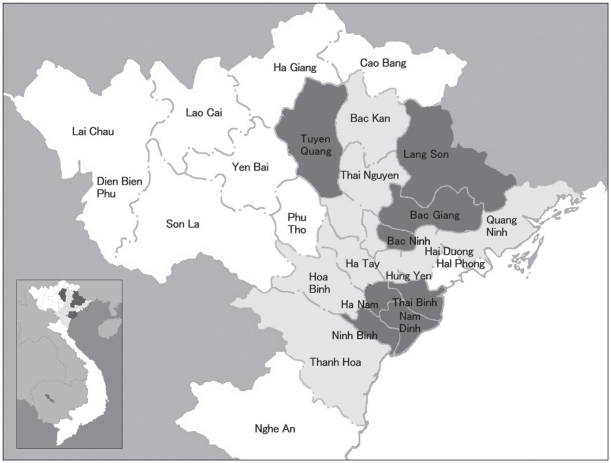
Map of study province. Grey and black areas show provinces in which the original educational programs were conducted in northern Vietnam. Black areas show the provinces where study sites were located for the present questionnaire survey.

The questionnaire survey was conducted in 8 general provincial hospitals where both the educational training and workshops were previously conducted: Bac Ninh, Bac Giang, Lang Son, Ninh Binh, Ha Nam, Nam Dinh, Tuyen Quang, and Thai Binh (Fig. 1). The average of number of beds in each hospital was approximately 600 and the average of number of staffs was approximately 200. Each hospital was the center of healthcare for a province. A total 334 study participants were selected by random sampling from the list of the staff in infectious disease department, intensive care unit, emergency department, and microbiology department in each hospital, and included physicians, nurses, laboratory technicians, pharmacists, and other healthcare professionals. All provinces in which the study hospitals were located have reported H5N1 epizootic outbreak among birds. 5 of the 8 provinces, including Ninh Binh, Ha Nam, Nam Dinh, Tuyen Quang, and Thai Binh, have reported human H5N1 patients. Respondents were divided into those 35 years of age and younger, 36 to 50 years, and over 50 years in order to determine behavioural and motivational differences between younger and older medical professionals.

### Original educational programs

Educational programs relating to H5N1 infection for medical providers were conducted for 20 hospitals in 18 provinces in northern Vietnam (Fig. 1) between 2008 and 2010. The original educational programs consisted of 2 parts: educational training and follow-up workshops. The training program was carried out by disseminating general scientific information and actual practical techniques and clinical methods relevant to the healthcare environment and H5N1 infection. All lecturers were medical doctors who had experience caring for H5N1 patients. The workshop was organized to reinforce knowledge and offer problem-solving discussion. People who participated in the programs and workshops were mostly physicians and laboratory technicians, but also included other medical professionals such as nurses and pharmacists.

### Survey Methodologies

The questionnaire survey was conducted between December 2010 and January 2011. A self-administered, structured questionnaire was created and delivered to study subjects in participating hospitals. The questionnaire was designed to assess KAP and was translated into Vietnamese. It collected information on demographics, information resources used for avian influenza, history of participation in educational programs, general knowledge, clinical attitude and practice, and motivation concerning caring for H5N1 patients. The pilot survey was conducted at Bac Kan General Provincial Hospital in April 2010 in order to develop the questions used in the present study.

All questions were either closed-ended or multiple choice. Some questions on clinical attitude and practice related to treatment for patients with suspected H5N1 were answered only by physicians. The variables and frequencies were assessed and compared for each healthcare professional group surveyed, including physicians, nurses, laboratory technicians and others.

The level of motivation was assessed according to 5 questions regarding concerns about treating H5N1 patients and the associated risks. To each question a maximum of 3 points was assigned to ‘agree,’ which presented positive thoughts concerning caring for H5N1 patients, 2 points were assigned to ‘undecided,’ and 1 point for ‘disagree,’ according to a 3-point Likert-type scale.

The study was approved by the institutional review boards of the Ministry of Health-Vietnam, Bach Mai Hospital and the National Center for Global Health and Medicine. Written informed consent was obtained from all study subjects.

### Statistical Analysis

Data from the surveys were double-entered using Microsoft Access 2007 (Microsoft, Redmond, WA, USA) and analyzed using SPSS ver. 19 (IBM, Armonk, NY, USA). For categorical variables, frequencies of profession, and age group were compared using the chi-square test and Fisher's exact test. For the determination of independent factors for motivation score, a step-wise selection method was used to select variables for multiple regression analysis. For all analyses, significance levels were two tailed, and a P value of <0.05 was considered to represent statistical significance.

## Results

### Characteristics and information resources for respondents

Out of 334 selected staffs in the 8 provincial hospitals (Fig. 1), a total of 326 (97.6%) respondents agreed to participate in the study and completed the questionnaire. The proportion of staff participated from each hospital was 12.5%. Backgrounds of respondents are listed in Table 1. Most of the respondents were physicians (64.7%), followed by nurses (18.1%) and laboratory technicians (16%). A total of 41% of respondents were aged 35 and younger, 42% were aged between 35 and 50 years. 47.5% of respondents previously attended both original training programs and follow-up workshops. Many respondents also previously attended educational programs which conducted by national or local healthcare departments, and/or by international health-related organizations (31.9% and 5.8%, respectively). Participation for International educational programs was more found on physicians than other professionals (p<0.001).

**Table 1:** Respondents' background.

	Physicians	Nurses	Laboratory technicians	Others	Total	p value
**No. of respondents (% of total)**	211 (64.7)	59 (18.1)	33 (10.1)	23 (7.1)	326 (100)	
**Male gender - No. (%)**	111 (52.6)	6 (10.0)	5 (15.2)	10 (43.5)	326 (100)	0.000
**Age - (years)**						
≤35	84 (39.8)	21 (35.6)	15 (45.5)	10 (43.5)	130 (39.9)	0.398
36–50	84 (39.8)	28 (47.5)	16 (48.5)	11 (47.8)	139 (42.6)	
≥51	43 (20.4)	10 (16.9)	2 (6.1)	2 (8.7)	57 (17.5)	
**Participation for educational programs relating to H5N1**						
Original educational programs*						0.171
Participated either training program or workshop	56 (26.1)	12 (20.3)	11 (33.3)	2 (8.7)	81 (24.8)	
Participated both training program and workshop	100 (47.4)	29 (49.2)	16 (48.5)	10 (43.5)	155 (47.5)	
**Educational programs other than original program**						
Local program†	74 (35.1)	15 (25.4)	6 (18.2)	9 (39.1)	104 (31.9)	0.137
International program‡	11 (5.2)	0 (0.0)	7 (21.2)	1 (4.3)	19 (5.8)	0.000
**Information resources (multiple choice) – No.(% of group)**						
Television	164 (77.7)	43 (72.9)	28 (84.8)	17 (73.9)	252 (77.3)	0.593
Radio	93 (44.1)	37 (62.7)	14 (42.4)	9 (39.1)	153 (46.9	0.060
Newspapers	131 (62.1)	28 (47.5)	24 (72.7)	8 (34.8)	191 (58.6)	0.007
Internet	119 (56.4)	27 (45.8)	11 (33.3)	10 (43.5)	167 (51.2)	0.052
Colleagues	127 (60.2)	40 (67.8)	22 (66.7)	14 (60.9)	203 (62.3)	0.695
Professional journals	79 (37.4)	23 (39.0)	8 (24.2)	4 (17.4)	114 (35.0)	0.123
Conferences and Educational programs	149 (70.6)	27 (45.8)	24 (72.7)	12 (52.2)	212 (65.0)	0.002
Others	5 (2.4)	1 (1.7)	0 (0.0)	1 (4.3)	7 (2.1)	0.002
**Attention to H5N1 treatment – No. (% of group)**						
I believe that H5N1 infection is likely to occur in my hospital.	184 (88.5)	50 (86.2)	33 (100.0)	23 (100.0)	290 (90.1)	0.245
H5N1 infection is a treatable illness.	201 (96.6)	58 (98.3)	33 (100.0)	20 (87.0)	312 (96.6)	0.062

*The original educational programs which were previously conducted by study investigators.

† Educational programs which were conducted by government or health department of province.

‡ Educational programs which were conducted my international heal-related organization such as the World Health Organization and Center for Disease Control and Prevention.

**Figure 2:**
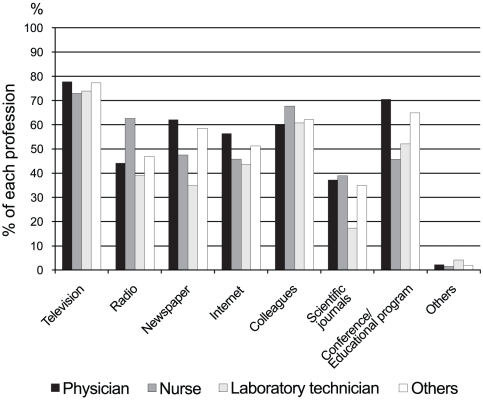
Information resource relating to H5N1 infection on each professional.

Information sources concerning avian influenza (H5N1) are listed in Table 1 and compared in Fig. 2. The most commonly reported information source for all respondents was television (77.3%), and was followed by conferences and educational programs (65.5%), colleagues (62.3%), and newspapers (58.6%). Conferences and educational programs were major information resources for physicians, and there was significant difference among professionals (p = 0.002). Colleagues were listed as information sources by over 60% of each professional group. Scientific journals were less listed as information sources, less than 40% of each profession, and there was no significant difference among the professionals. (p = 0.123).

Concerning the attention relating to H5N1 infection, 90.1% of respondents believed that H5N1 patients were likely to present for treatment in their hospital, and 96.6% of respondents were aware that H5N1 infection is a treatable illness.

### Knowledge associated with H5N1 infection for hospital-based healthcare professionals

Knowledge associated with H5N1 infection among healthcare professionals is shown in Table 2. 93.9% of laboratory technicians provided correct answers to questions about whether H5N1 infection is transmitted by eating uncooked infected poultry and by eating uncooked eggs, while correct answers for those 2 questions were provided by 88.2% and 86.3% of physicians, respectively (p = 0.040). Almost all physicians, nurses, and laboratory technicians (93.4%, 93.2%, and 100%, respectively) correctly answered that H5N1 infection is transmitted by touching infected poultry. Almost 100% of respondents understood that personal protective equipment (PPE) was necessary for contact with patients with suspected H5N1.

**Table 2:** Knowledge associated with H5N1 infection.

	Physicians	Nurses	Laboratory technicians	Others*	Total	p value
No. of subjects who obtained correct answer (% of each profession)	211 (64.7)	59 (18.1)	33 (10.1)	23 (7.1)	326 (100)	0.000
H5N1 infection is a treatable illness.	201 (92.6)	58 (98.3)	33 (100.0)	20 (87.0)	312 (95.7)	0.062
H5N1 infection is transmitted by eating uncooked infected poultry.	186 (88.2)	43 (72.9)	31 (93.9)	19 (82.6)	279 (85.6)	0.040
H5N1 infection is transmitted by eating uncooked infected eggs.	182 (86.3)	47 (79.7)	31 (93.9)	18 (78.3)	278 (85.3)	0.149
H5N1 infection is transmitted by touching infected poultry.	197 (93.4)	55 (93.2)	33 (100.0)	21 (91.3)	306 (93.9)	0.860
Early initiation of oseltamivir administration is necessary for treating H5N1 patients.	189 (89.6)	54 (91.5)	27 (81.8)	17 (73.9)	287 (88.0)	0.319
Personal protective equipment is necessary for contact with patients suspected of having H5N1.	207 (98.1)	59 (100.0)	33 (100.0)	23 (100.0)	322 (98.8)	1.000

*Others includes pharmacists and hospital administrators.

### Clinical attitudes and practices of physicians concerning H5N1 patient treatment

Clinical attitudes and practices associated with treatment of H5N1 patients were assessed only for physicians and compared by age groups, and results are shown in Table 3. Contact history with sick and dead poultry was the most frequent response to suspecting H5N1 infection for all age groups followed by high fever. Suspecting H5N1 infection in patients presenting with an infiltrative shadow was listed by 14.3%, 27.4%, and 34.9% of physicians, respectively, and there was a significant difference between age groups (p = 0.021). Almost all physicians suspected H5N1 infection if patients visited the hospital with flu-like symptoms during the same period when an H5N1 epizootic outbreak among birds was happening in their locale. Also, almost all said that they would ask patients if they had contact with infected poultry and they said they pay attention to the local situation with respect to H5N1 epizootic outbreak among birds. 96.2% of physicians reported asking patients to wear a mask if the patients are coughing. Older aged physicians more demonstrated patients how to prevent infection using a mask and gloves than aged 35 yr. and less (p = 0.049). 15.6% of physicians waited to administer oseltamivir until receiving positive results for H5N1 virus infection by a real-time reverse-transcriptase-polymerase-chain-reaction (RT-PCR) assay. Over 90% of physicians reported consulting an expert who has ever experienced caring H5N1 patients when they received H5N1 patients including suspected cases.

**Table 3:** Physicians' clinical attitude and practice for treating H5N1 patients.

Age group (years)	≤35	36–50	≥51	Total	
	84 (39.8)	84 (39.8)	43 (20.4)	211 (100)	p value
Variables - No. (% of group)					
What are important symptoms and signs for you to suspect H5N1 infection?					
High fever	63 (75.0)	62 (73.8)	26 (60.5)	151 (71.6)	0.192
Dry cough	11 (13.1)	8 (9.5)	8 (18.6)	27 (12.8)	0.348
Contact history with infected poultry	81 (96.4)	79 (94.0)	42 (97.7)	202 (95.7)	0.744
Infiltration shadow on chest radiograph	12 (14.3)	23 (27.4)	15 (34.9)	50 (23.7)	0.021
Other symptoms	12 (14.3)	17 (20.2)	12 (27.9)	41 (19.4)	0.180
Do you pay attention to the local situation of epidemic outbreaks among birds?	82 (98.8)	83 (98.8)	42 (100.0)	207 (99.0)	1.000
When epidemic outbreaks among birds happen, do you suspect H5N1 infection for patients with flu-like symptoms?	83 (98.8)	81 (96.4)	42 (97.6)	206 (97.6)	0.689
Do you wear PPE* when you see suspected H5N1 patients?	82 (97.6)	81 (96.4)	41 (97.6)	204 (97.1)	1.000
Do you ask patients to wear a mask if they are coughing?	79 (94.0)	80 (96.4)	42 (100.0)	201 (96.2)	0.326
Do you demonstrate to patients how to prevent H5N1 infection using mask and gloves?	74 (88.1)	81 (96.4)	40 (95.2)	195 (92.9)	0.049
Do you wait to administer oseltamivir until getting RT-PCR† test results for a patient with suspected H5N1?	14 (16.7)	13 (15.7)	6 (14.3)	33 (15.6)	0.789
Do you pay attention to the availability of oseltamivir?	51 (60.7%)	65 (77.4%)	30 (69.8)	146 (69.2)	0.210
Do you consult with experts who have experience caring for H5N1 patients if you receive a patient with suspected H5N1?	75 (90.4)	77 (91.7)	37 (88.1)	189 (90.4)	0.776

*PPE: personal protective equipment.

† RT-PCR: real-time reverse-transcriptase-polymerase-chain-reaction.

### Motivation for caring for H5N1 patients among hospital-based medical providers

Responses for the 5 questions related to evaluation of motivation for caring for H5N1 patients were compared among professionals (Table 4). Almost all respondents (98.5%) reported feeling glad to have the chance to contribute to society by treating H5N1 patients. All laboratory technicians reported accepting the risks of H5N1 infection because of their profession, and 81.0% of physicians and 83.0% of nurses reported accepting such risks. Also, almost all physicians, nurses, and laboratory technicians reported agreeing to care for H5N1 patients and reported that they were not afraid of becoming infected from H5N1 patients.

**Table 4:** Professional motivation relating to care H5N1 patients.

	Physicians	Nurses	Laboratory technicians	Others*	Total	p value
No. of subjects (% of total)	211 (64.7)	59 (18.1)	33 (10.1)	23 (7.1)	326 (100)	
I accept the risks of H5N1 infection because of my profession.	171 (81.0)	49 (83.1)	33 (100.0)	21 (91.3)	274 (84.0)	0.036
I am glad if I have a chance to contribute to society by treating H5N1 patients.	208 (98.6)	57 (96.6)	33 (100.0)	23 (100.0)	321 (98.5)	1.000
I should care for H5N1 patients.	200 (94.8)	3 (5.1)	4 (12.1)	0 (00)	18 (5.5)	0.263
I am not afraid to become infected from H5N1 patients.	177 (83.9)	49 (83.1)	33 (100.0)	21 (91.3)	280 (85.9)	0.064
I will not resign if I need to treat a H5N1 patient.	31 (14.7)	7 (11.9)	1 (3.0)	3 (13.0)	42 (12.9)	0.422

*Others includes pharmacists and hospital administrators.

Concerning motivation scores, out of 15 possible points, physicians, nurses, laboratory technicians and others reported 13.9, 13.9, 14.7, and 14.4 points, respectively (Table 5). All healthcare professionals presented high motivation scores, and laboratory technicians presented the highest motivation scores (p = 0.001).

**Table 5:** Professional motivation scores associated with H5N1 infection among healthcare profession.

Healthcare profession	Mean	Standard deviation	Range
Physician	13.9	1.66	7–15
Nurse	13.9	1.56	7–15
Laboratory technician	14.7	0.82	12–15
Others*	14.4	1.08	11–15

P = 0.001^†^.

* Others includes pharmacists and hospital administrators.

†1 way-ANOVA using Welch's test for four independent samples.

Factors associated with scores concerning professional motivation for caring for H5N1 patients were assessed using multiple regression analysis by independent factors of background of respondents and are shown in Table 6. Healthcare profession, the respondents' work place (hospital), age group, and attendance at an original educational program were evaluated as factors that were seen to have an influence on the motivation for caring H5N1 patients. Attendance at educational programs by international health-related organization was associated with increased professional motivation.

**Table 6:** Factors influencing motivation to care for H1N1 patients using multiple regression analysis.

Factors	Coefficients	Standard error	t value	p value	95% confidence interval
Constant	13.955	0.317	44.030	0.000	13.332–14.579
Professionals*	0.210	0.090	2.319	0.021	0.032–0.387
Work place (hospital)†	0.129	0.037	3.435	0.001	0.055–0.202
Age group‡	−0.333	0.118	−2.834	0.005	−0.565–−0.102
Attend educational programs by international organizations§	0.882	0.363	2.431	0.016	0.168–1.596
Attend original educational programs¶	−0.225	0.102	−2.218	0.027	−0.425–−0.025

*Professionals includes hospital based physician, nurse, laboratory technician, and other healthcare providers.

† Hospital denotes the provincial hospitals where the respondents worked: Bac Ninh, Bac Giang, Lang Son, Ninh Binh, Ha Nam, Nam Dinh, Tuyen Quang, and Thai Binh.

‡ Age groups were 35 years of age and younger, 35 to 50 years, and over 50 years.

§ Educational programs which were conducted my international heal-related organization such as the World Health Organization and Center for Disease Control and Prevention.

¶ The original educational programs which were previously conducted by study investigators.

## Discussion

The present study revealed a high level of knowledge and appropriate clinical behaviours relating to H5N1 infection in humans among hospital based medical providers in a high risk area in northern Vietnam, even though only approximately 50% of respondents had received relevant educational programs. In addition, nearly 100% of respondents reported that they were glad to treat H5N1 patients and presented high professional motivation. Independent factors which influenced a high motivation to care for H5N1 patients were healthcare profession, the respondents' work place (hospital), age group, and history of participation in educational programs. The participation histories for educational programs conducted by international health-related organizations were specifically associate with professional motivation.

Although previous studies have demonstrated the importance of early initiation of treatment for treating H5N1 patients [2], [3], [6], [7], [8], [9], knowledge, practical behaviours and attitude of medical providers who implement treatments for H5N1 patients have not been reported from Vietnam. The subjects of the present study were hospital based healthcare professionals in high-risk H5N1 areas in northern Vietnam. In past years, we have developed the networking programs among hospitals and clinicians which connected tertiary care hospitals and central hospitals in 18 provinces for purposes of exchanging the scientific information, consultation, and transferring patients. Despite this, face-to-face discussions relating to H5N1 infection within and between provinces have been limited. In the present study, conferences and educational programs were the second highest sources of information on H5N1 (65%) following television (77.3%). Colleagues were also commonly listed as a source of information (62.3%). Scientific journals were reported as sources by only 35.0% of total respondents, and by 37.4% of physicians. These results indicated that medical providers outside of metropolitan areas receive professional and scientific information mostly from conferences and educational programs. Attending such meetings can offer opportunities to share information and have face-to-face discussions among colleagues and friends. It is therefore suggested that educational programs play an important role in providing professional information, especially for providing practical knowledge and technical skills which medical providers can use for treatment of H5N1 patients. Under the situation of relatively small number of H5N1 patients, sharing experiences and getting practical information from medical providers who have experienced treating H5N1 patients is crucial. Although scientific journals are important for collection of formal data and evidence, only a small percentage of those surveyed responded that scientific journals were sources of information on H5N1. This suggested that scientific information concerning H5N1 should be included in educational programs.

The results of the present study indicate that a high number of respondents had an understanding of H5N1 infection and a high level of knowledge of the disease. A high level of positive clinical attitudes and knowledge/use of appropriate practices for treating H5N1 patients was reported by physicians. Since many diseases present with high fever, contact history with dead/sick poultry is obtained as a unique diagnostic indicator for H5N1 infection. However, an infiltration shadow on chest radiograph was not reported by many physicians as a factor for suspecting H5N1 infection, even though observation of pneumonia is crucial since H5N1 infection presents rapid progression to pneumonia. The RT-PCR test for H5N1 was only available in limited facilities in Vietnam, but 15.6% of physicians said that they delay oseltamivir administration until they receive a positive RT-PCR result. This may be a primary cause of delay in antiviral treatment, even though providers understand the importance of early medical intervention for treating H5N1 patients. These issues (use of chest radiographic results and pre-emptive treatment prior to RT-PCR confirmation) should be included the further educational programs.

All study subjects presented high professional motivation for caring for H5N1 patients. A general fear of infection by transmission was not found (Table 4). This may result from an understanding of the importance of wearing PPE to prevent infection from H5N1 patients (Tables 2 and 3). High professional motivations contribute to active care for patients with highly pathogenic influenza infection. Also, the subjects' understanding that H5N1 patients were likely to present in their work place, and their confidence that H5N1 infection is a treatable illness, may have contributed to their high motivation (Table 1). The motivations score were influenced by professions, work place (hospital), age group, and attendance at educational programs, including both local and international programs. The results suggested that, independent of the provider, educational programs contribute to professional motivation for treating H5N1 patients. On the other hand, over 90% of physicians said that they seek consultation from physicians who have treated H5N1 patients when H5N1 (suspected) patients are admitted at their hospital (Table 3). This result indicates that physicians need to have practical suggestions once they receive H5N1 (suspected) patients. This indicated that the networking is necessary beyond hospitals and provinces before receiving H5N1 patients.

The present study was conducted among limited subjects who worked in provincial hospitals in which H5N1 original educational programs were previously conducted. The results may differ for KAPs for hospital based medical providers in other provinces which have never had such educational programs. The high response rate (97.6%) indicated that the respondents represented the actual hospital staff, and their encouragement of participation in the study was greatly respected and appreciated.

Educational programs which were previously conducted in Vietnam appear to have contributed to the KAPs and high motivation which allow early diagnosis and early initiation of treatment for H5N1 patients. Additional educational programs created with the results of the present study are needed - specifically, increased education related to the use of chest radiology results and a willingness to treat in the absence of RT-PCR results (when immediate testing is unavailable) may further increase effective treatment. Furthermore, strengthening of the network for sharing scientific information and experiences concerning H5N1 is important. Education and networking may contribute to enhancing roles of medical providers in H5N1 high-risk areas, which may result in a reduction of mortality from H5N1 infections.
